# Physiotherapy assessment of breathing pattern disorder: a qualitative evaluation

**DOI:** 10.1136/bmjresp-2022-001395

**Published:** 2023-01-10

**Authors:** Lizzie Grillo, Anne-Marie Russell, Harriet Shannon, Adam Lewis

**Affiliations:** 1 National Heart and Lung Institute, Imperial College London, London, UK; 2 Department of Physiotherapy, Royal Brompton and Harefield Hospitals, London, UK; 3 Exeter Respiratory Innovations Centre, University of Exeter, Exeter, UK; 4 Physiotherapy, Institute of Child Health, Great Ormond Street Hospital, University College London, London, UK; 5 Department of Health Sciences, Brunel University London, London, UK

**Keywords:** perception of asthma/breathlessness

## Abstract

**Objectives:**

To explore physiotherapists’ opinions of physiotherapy assessment of Breathing Pattern Disorder (BPD).

**Methods:**

Qualitative study using focus groups (FGs) with reflexive thematic analysis and survey methods. The survey was distributed via social media and email to UK specialist physiotherapy interest groups. Two FGs, conducted in different settings, included physiotherapists based in hospital outpatients/community, private practice and higher education.

**Results:**

One-hundred-and-three physiotherapists completed the survey. Respondents identified a lack of consensus in how to define BPD, but some agreement in the components to include in assessment. Fifteen physiotherapists participated in the FGs. Three themes emerged from FG discussions: (1) nomenclature and language of breathing, (2) BPD and breathlessness and (3) The value of assessment of breathlessness.

**Conclusion:**

The inconsistent nomenclature of dysfunctional breathing pattern impacts assessment, management and understanding of the diagnosis. Clarity in diagnosis, informing consistency in assessment, is fundamental to improving recognition and treatment of BPD. The findings are useful in the planning of education, training, future research and guideline development in BPD assessment.

**WHAT IS ALREADY KNOWN ON THIS TOPIC**

- Breathing pattern disorder is an important condition. It is associated with significant morbidity and can be treated with physiotherapy. Limited evidence exists regarding how best to assess and recognise BPD, which may limit the opportunity for patients to be referred promptly to services to receive the care they need.

**WHAT THIS STUDY ADDS**

- The first qualitative clinician-focused investigation of breathing pattern disorder that includes in-depth evaluation of physiotherapists’ opinions of breathing pattern assessment and provides a practical summary of the important components of its assessment to be used in clinical practice

**HOW THIS STUDY MIGHT AFFECT RESEARCH, PRACTICE OR POLICY**

- The study provides a clear description of the need for consistency around terms used and approaches to the assessment of breathing pattern disorder. The themes identified in this study could help to direct future education, training and guidance for this condition and help underpin the development of future research into this area.

## Introduction

In health, and at rest, human respiration is largely achieved through the subconscious rhythm of breathing at a comfortable tidal volume. Increases in both the rate and depth of breathing are triggered by temporary, physiological responses to stimuli, including sympathetic activity, exertion or anxiety.^1^ Disorders in breathing pattern are known most commonly as breathing pattern disorder (BPD) or dysfunctional breathing. Individuals with BPD tend to breathe in a manner that is disconnected from their respiratory or metabolic requirements,^2^ in some cases leading to decreased arterial partial pressure of carbon dioxide through hyperventilation.^3^ They mayalso experience breathlessness, air hunger and limitations in function.^4^ Its pathogenesis remains unclear, but probably comprises an interplay between biomechanical and biochemical stimuli and psychopathological cognitive factors.^5^ The condition may be present in the absence of respiratory disease (‘primary’ BPD) or it may accompany another respiratory disorder, commonly asthma^6^ (‘secondary’ BPD) or, more recently, as a cardinal feature of long COVID-19.^7^

The manifestations of BPD are readily misinterpreted—by both patients and clinicians—as those of asthma or similar obstructive and restrictive conditions.^8^ This leads to prescriptions of medications that are neither required nor effective, including inhaled or oral steroids.^1^ Furthermore, BPD frequently amplifies other respiratory conditions, increasing the likelihood of excess prescriptions and associated misuse of prescribing budgets.^1 9^ In COVID-19 follow-up clinics, a high proportion of patients reported of disproportionate breathlessness, alongside symptoms of fatigue and decreased exercise tolerance.^10^ While most post-COVID-19 clinics request a physiotherapy assessment of BPD as a key management step, diagnosis is hampered by the absence of universally accepted terminology or method of assessment.^11^ Moreover, BPD does not always present in the same way, a heterogeneity that may further lead to inconsistent assessment.^12^ Various assessment tools can be used to assess BPD but many do not capture the core components across all types of presentation.^13^

Due to the apparent complexity of BPD, it is essential to increase our understanding of how to approach assessment. A consistent assessment will help to recognise the disorder and ensure appropriate treatments are offered to patients. This paper aims to further our understanding of BPD by exploreing expert physiotherapists’ perspectives on BPD assessment.

## Methods

The study consisted of two parts. An electronic survey was developed and distributed, and two semistructured focus groups (FGs) were undertaken. The electronic survey was distributed in October 2019. The specific objectives were:

1. To evaluate clinicians’ preferred descriptors for this condition.
2. To describe components frequently included in the assessment of BPD (objective and subjective).

This 24-item survey was developed in collaboration between authors (LG and HS) and a specialist physiotherapist working in BPD (see online supplemental file 1) using SurveyMonkey. Survey items included components of assessments completed within the physiotherapy department and questionnaires available including the Breathing Pattern Assessment Tool (BPAT)^14^ and Nijmegen Questionnaire (NQ).^15^ The items were selected and informed by clinical expertise and included the common components of physiotherapy assessments in usual accepted practice, with reference to published guidance where physiotherapy assessment had been described.^14 16 17^ The penultimate survey draft was peer reviewed. The survey was piloted by two physiotherapists in a tertiary referral centre. The final version was distributed via UK physiotherapy professional networks, including the Association of Chartered Physiotherapists in Respiratory Care (ACPRC), Physiotherapy for Breathing Pattern Disorders UK (a specialist clinical interest group) and other regional groups via email and social media platforms. Email reminders were sent after 1 month and a week prior to close of the survey. Participation was voluntary and consent was gained at the start of the survey (see online supplemental file 1). A convenience sample was the most pragmatic approach.

10.1136/bmjresp-2022-001395.supp1 Supplementary data

Following completion of the survey, LG and AL facilitated two semistructured FGs, to explore physiotherapists’ understanding and perceptions of the assessment of BPD. A topic guide was developed by LG/AL, informed by the survey design and results (see online supplemental file 2). Open-ended questions were included within the FGs. Participants were screened by LG. Inclusion criteria included (1) qualified physiotherapist (Agenda for Change Band 6 (specialised physiotherapist with approximately 2–5 years of experience) and above), (2) actively treating patients with BPD (at least one patient a week), (3) able to attend an FG (prepandemic). All participants received a participant information sheet and gave informed consent. FGs were audio-recorded. The researcher’s reflections were also recorded (see online supplemental file 2).

10.1136/bmjresp-2022-001395.supp2 Supplementary data

### Patient and public involvement

The research question emerged from informal discussions with patients whom LG treated for BPD. Patients expressed frustration that their condition remained under-recognised and expressed concern over delays in diagnosis. Furthermore, LG facilitated a patient and public involvement day involving patients with BPD. Discussions focused on the assessment and treatment of BPD as well as priority research topics. Feedback included: ‘This is a condition all in itself, not just one linked to asthma or psychology, therefore it needs describing more clearly’ and ‘This is a condition that, if untreated, could lead to a lot of wasted money on treatments and investigations, this makes it very important’. This research was completed as part of NIHR (National Institute of Health and Care Research) supported predoctoral training informing a Clinical Doctoral Research that will comprehensively explore the assessment of BPD. We did not formally include patients as research partners in this aspect of the study but recognise the importance of this in future work.

### Data analysis

Survey information was collated and displayed as percentages. Individual answers from incomplete surveys are also included. Free text was analysed thematically. Interviews were transcribed verbatim by LG and transcripts were reviewed by LG and AL. LG conducted a reflexive thematic analysis.^18 19^ AL participated in the analysis to challenge and further develop interpretation following joint review of transcripts and preliminary themes.

## Results

### Online survey

Of the 103 responses to the survey, 98 were completed in full (one response missing, n=2; 2 responses missing, n=3). 93% of respondents were working in the UK; 75% (n=77) at band 7 level (highly specialised physiotherapist, usually with 5+ years of clinical experience) or above. Most respondents worked in an outpatient setting at least some of the time, with 64% (n=66) working in inpatients as well. Forty-five per cent (n=46) saw at least 1–5 patients with BPD per week, with a further 35% (n=36) seeing over five patients per week.

**Figure 1:**
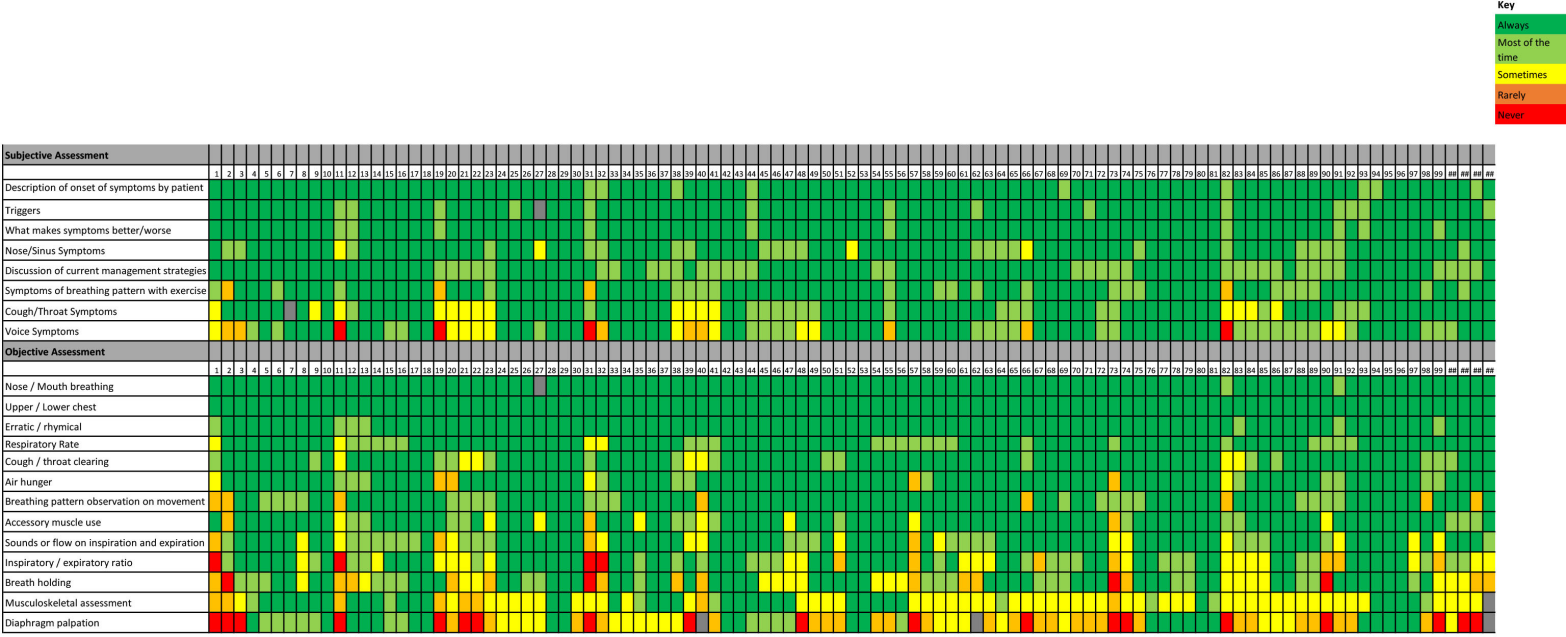
Vild chart to show frequency of subjective and objective components used by physiotherapists in the assessment of BPD. BPD, breathing pattern disorder.

There were discrepancies in the preferred term used to describe the condition, although the majority used either BPD (43%) or dysfunctional breathing (39%) (table 1). Aspects of the subjective and objective assessment were consistently used (figure 1). Differences were noted in cough/throat and voice symptoms in the subjective components and inspiratory and expiratory ratio in the objective components. Musculoskeletal assessment and diaphragm palpation showed more variability in frequency of use. Respondents felt confident in assessing BPD with the median Visual Analogue Scale score for confidence being 8/10 (ranging from 6/10 to 10/10). There was variable use of patient-reported outcome measures, objective outcome measures and observed assessments (see table 1).

**Table 1:** Physiotherapists’ preferred term for this condition and frequency of subjective and objective/observational outcomes used

Preferred term to describe condition (n=103)	Percentage (n)
Breathing pattern disorder	43% (n=44)
Dysfunctional breathing	39% (n=40)
Breathing pattern dysfunction	14% (n=14)
Hyperventilation	4% (n=4)
Incomplete response	1% (n=1)

Patient reported outcome measure (n=103)	Percentage
Short evaluation of breathing questionnaire^30^	14% (n=14)
Dyspnoea 12^29^	35% (n=36)
BORG (RPE)^39^	46% (n=47)
Nijmegen questionnaire^40^	89% (n=92)
Hospital anxiety and depression	17% (n=18)
Other included PHQ, GAD-7	4 responses

Objective/observational tool (n=101)	Percentage
Breath hold^31^	61% (n=62)
End tidal C02	0% (n=0)
Exercise Ax (eg, 6MWT)	39% (n=39)
Manual Ax of respiratory motion^32^	11% (n=11)
Breathing pattern assessment tool^14^	58% (n=59)
Other included lung function	1 response
Incomplete response	1% (n=2)

BORG (RPE), The Borg Rating of Perceived Exertion (RPE); GAD-7, Generalised Anxiety Disorder Assessment; 6MWT, six-minute walk test; PHQ-9, Patient Health Questionnaire.

### Focus groups

Twenty-two participants who completed the survey registered interest in joining the follow-up FG study. Fifteen were selected to participate in one of two FGs. Reasons for non-participation included the date not being suitable, or time constraints. FG-1 included eight participants (one man) and FG-2 seven participants (two men). The FGs lasted 78 and 93 min, respectively, and were audio recorded. Thematic analysis of the transcripts identified three main themes: nomenclature and language, BPD and breathlessness and the value of assessment. This final theme was subdivided into further subthemes, including; (a) components of assessment, (b) validation of symptoms and (c) specialist skills. Illustrative quotations from FG participants included are seen in box 1.

Box 1 **Focus group quotes**

**Nomenclature**

‘There are so many different words for the same thing…it is so confusing which must impact how the condition is perceived.’ (R4)

‘I think Dysfunction can feel like a blaming term, I don’t think it is popular with patients…this can sound negative, and patients can think it is their fault’ (R2)

I use dysfunctional breathing, but I am quite happy to switch between breathing pattern disorder. I think hyperventilation is an element within it which not everybody has (R14)

‘So, I think the terminology for me, in my experience with patients, doesn’t matter as much as the fact that someone is acknowledging what they are feeling and validating the symptoms they are experiencing’ (R6)

‘The negative terminology vs the lack of definition is a frustration as it can devalue the experience and the condition greatly’. (R5)

‘I don’t know that patients actually want a diagnosis or a label as such. If you just tell them, ‘Well actually, it is as simple as your breathing pattern has gone wrong,’ they all seem to understand that’ (R13)

‘I think it is frustrating, the terminology. It doesn’t help does it, in us trying to help diagnose, doing research, you know having outcome measures, when we sort of don’t even have this start point’ (R7)

**Language**

‘I think that is quite a key component for your journey into treatment. If you don’t get that right, and you don’t get that connection and that validation or understanding, if you go straight into putting into boxes…’ (R9)

‘And I think, like in all of the literature, all of the stuff around dysfunctional breathing, it is all based on like expert opinion……then almost no patient voice at all. Like there is almost no qualitative research on what the experience of having a dysfunctional breathing pattern is like. We just measure it with like lists of symptoms or a very biomechanical focus’ (R12)

‘It becomes kind of a shorthand for some quite negative narratives about like difficult patients who are anxious. Like, anxiety isn’t just a thing, it is a thing that needs to be treated and you need to manage it in a way that is more than doing a couple of days of CBT training’ (R9)

**Breathing patten dysfunction and breathlessness**

‘I feel like the feeling of breathlessness is very different, and it is more transient for BPD than it would be for someone with COPD. It is about managing it with a COPD condition, whereas with disordered breathing it is more trying to get to the root cause and fixing it’ (R2)

‘I am not sure that many of our patients are able to describe what they are feeling, it is only once we describe BPD to them that they feel their symptoms are validated.’ (R8)

‘But you know, they have come through such a medical process that sometimes you get to the end and actually it is not that medical, and they need that unpicking and they need releasing from that diagnosis element, from that pathology’ (R14)

‘I think it is that breathlessness that doesn’t make sense and that is not accepted maybe. It doesn’t correlate with the objective tests that have been done’ (R8)

**The value of assessment**

*Diagnosis and validation of symptoms*

‘The patients really like how much we validate their symptoms, and just having it explained to them, means so much to them and it is such an important thing to capture’ (R14)

‘I guess what you are curious about at the beginning of your assessment is, is this breathlessness in excess of something that is existing, or in the absence of something there’ (R16)

*Components of Ax*

‘The patient’s own description of the symptoms can be really insightful as quite often they do not understand their symptoms or make connection without the direction from the physiotherapy assessment.’ (R11)

‘It is a privilege to have the time to fully understand these symptoms, often such time is absent in nursing and medical appointments.’ (R14)

**Subjective Ax**

‘Yeah, I think the subjective is probably most important, you are trying to understand the long and winding road that they have taken to get to you’ (R4)

‘I think it is about recognising (psychology), and very solidly knowing your boundaries, and it is about knowing when you have reached the edge of your boundaries and when to hand over. Because I used to keep people on far too long when in actual fact it wasn’t their breathing that was the problem anymore, it was the psychology behind it’ (R11)

*Observational/objective*

‘There is often an impact of being watched or putting hands on the patient- important to look at breathing in less obvious ways and not just when the patient is aware of it’. (R10)

*Diaphragm*

‘The diaphragm could be at a mechanical disadvantage due to factors including abdominal tension, upper chest predominant breathing and breathing at a higher lung volume (if a patient was not optimising their expiratory time)’ (R2)

‘The diaphragm is moving. The fact that you may be holding your thorax or your abdominal compartment more tightly and you see less movement, doesn’t mean that the diaphragm isn’t actually moving’. (R7)

‘No, it isn't weak, it’s that the upper chest is being overused’ (R7)

*Assessment tools*

‘You know, there is a long way to go before we are in a position to say, ‘These are valid outcome measures, they are reproduceable across many different conditions etc.’ (R13)

‘I find for me, using the BPAT is very flexible with my different patient groups. I find that particularly helpful…………to validate where you feel that the biggest problem is’ (R2)

‘I can see why the structure of some of those things gives them a sort of foundation to sort of start building stuff on. Helping you to say, ‘I am going to look at these things.’ (R1)

‘Then I think the BPAT combined with kind of a hands on assessment is really the one where we are actually able to then quantify the problems that we are seeing, and I think there is something very helpful in feeding back to our team where we can say, ‘Here was their breathlessness scale, and here was my markers in a BPAT. We did treatment and now it is this.’ (R16)

*Exercise assessment*

‘They have been so activity avoidant that they are just deconditioned as well, and that is an additional thing that you are treating. You can only do that once their breathing pattern is ready for that essentially’. (R5)

**Specialised skills**

‘Basic skills in breathing pattern observation are not always given the adequate time and perhaps presented as a special skill’. (R4)

‘You only get to that point where you can really unpick everything if you have had a lot of support, and training, and exposure to these patients’ (R6)

#### Nomenclature and language

Participants expressed frustration around the nomenclature of BPD and would value consensus on the meaning of BPD. There was a tension individually and across different groups of clinicians as to the most appropriate term. This inconsistency risked diminishing the importance of the condition, leading to confusion over the diagnosis.

There was a variation of the terms used by the medical profession and those preferred by patients. The term ‘dysfunctional’ was used more readily by clinicians/medical teams. There was a strong assumption that the word ‘dysfunction’ was unpopular with patients.

‘The negative terminology versus the lack of definition is a frustration as it can devalue the experience and the condition greatly’. (R5)

For some, the terminology did not matter, so long as the diagnosis was explained well to the patient. The inclusion of the word ‘pattern’ was felt to be important as it describes objective changes in the patient’s presentation.

One of the challenges discussed was undoing the negative assumptions created in the language and explanations of BPD used by clinicians. Frequent associations between BPD and psychological or psychiatric problems were reported to potentially create further diagnostic challenge. This was possibly due to BPD being used as a negative label or not being given the status of a ‘real diagnosis’ by some referring clinicians. The tension between the physiological and/or psychological causes of symptoms often created negative language impacting symptoms where inpatients labelled as ‘trickier’ experienced devaluation of their BPD. Additionally, clinicians were aware of how patients often seemed uncomfortable when their symptoms were attributed to anxiety, depression of other psychological factors.

#### BPD and breathlessness

Detailed discussion was developed around what BPD was and how it presented within a broader description of breathlessness. BPD most often included a description of feeling out of breath (either self-reported by the patient or observed by others). BPD was different to the physiological breathlessness caused by a gas exchange impairment or a mechanical restriction or obstruction to breathing asin conditions such as chronic obstructive pulmonary disease (COPD) or interstitial lung disease.

I am not sure that many of our patients are able to describe what they are feeling, it is only once we describe BPD to them that they feel their symptoms are validated. (R8)

BPD presentation was often discordant with what one might expect to see. There was also recognition that patients could have heterogeneous symptoms, some clearly linked to their breathing, but other symptoms lacking such an obvious connection. Often BPD was clearly identified by a clinician in the assessment, but the label of BPD itself meant little to the patient until the symptoms and their potential impact were explained to them.

#### The value of assessment

The FG discussion described the importance of assessment in diagnosing this condition, to ensure patients could access the right treatments and services to optimise healthcare and outcomes. Additionally, this diagnosis validated the condition to the patient. Discussions also described the power of the assessment as therapy itself.

### Components of assessment

The FG expanded on the survey findings, detailing the importance of social, emotional, occupational and family impacts during the subjective patient assessment. FGs discussed the assessment of sleep quality and evaluation of the emotional components of breathing integral to this subjective examination. These were aspects of the assessment not covered in the survey.

All participants in the FG described the importance of having enough time in a BPD assessment to fully understand the symptoms as experienced by the patient. This was integral to the ability to diagnose ‘BPD’ and to help the patient understand the role of therapy. Clinicians in the FG expressed a need for 45–60 min for the assessment, with any less potentially ineffective. This corroborates the survey results in relation to optimal length for the initial contact with a patient (see online supplemental files).

It is a privilege to have the time to fully understand these symptoms, often such time is absent in nursing and medical appointments (R14).

### Observational/objective assessment

While hands on assessments were deemed important, participants noted that during such an assessment of breathing, patients often respond differently, altering their breathing pattern. Respiratory rate, nose versus mouth breathing, upper chest versus lower chest and whether breathing was erratic or rhythmical had high importance, reflecting survey results. In the FG, inspiration and expiration timing were important to determine the inspiratory/expiratory ratio. These components were identified as valuable to teaching self-assessment, an important therapeutic strategy.

There is often an impact of being watched or putting hands on the patient- important to look at breathing in less obvious ways and not just when the patient is aware of it (R10).

#### The diaphragm

There were some differences of opinion on the importance of, and how to assess or treat, the diaphragm. The survey suggested that diaphragm palpation was unpopular due to the likelihood of discomfort and the additional difficulties of palpation in patients with a Body Mass Index >30. The relevance of this in assessment, discussed in the FGs, centred on the role of the diaphragm in BPD, for which there were differing opinions. These included the possibility of the diaphragm being at a mechanical disadvantage due to tension in other muscles of the abdomen or thorax versus whether the diaphragm is weak causing impaired movement. There was a debate among the experts as to how to assess diaphragm function and its importance as a therapeutic strategy in BPD. There was agreement over the importance of assessing the musculoskeletal system due to the interrelation between breathing, and body tension and movement.

#### Assessment tools

Assessment tools were an important component of assessment. Despite the NQ not being validated for use in all types of BPD, FG discussion suggested that the symptoms and scores of patients with BPD could be elevated and that this was a useful tool to identify symptoms. The BPAT and Breath Hold (BH) were thought to have particular value when the assessor may have less experience and to help quantify improvements after treatments.

I guess you are trying to be as systematic with outcome measures as possible while acknowledging the complexity of the condition (R9).

### Exercise assessment within BPD

Assessment of BPD with exercise was not thought to be essential for all initial assessments of BPD due to time constraints. Different experiences were reported; for some clinicians, problems with exercise BPD could not be predicted from an assessment at rest, and for others resting BPD treatment was always important as resting breathing pattern optimisation was key prior to exercise assessment. Such heterogeneity in presentation made some feel this was an important area to assess, particularly that decreased function and its impact on quality of life was a common report from patients referred with BPD.

They have been so activity avoidant that they are just deconditioned as well, and that is an additional thing that you are treating. You can only do that once their breathing pattern is ready for that essentially (R5).

### Specialist skills

It was felt that assessment of BPD and breathlessness should be part of every cardiorespiratory physiotherapist’s assessment. Education about assessment of normal breathing and the impact of pattern abnormalities should be taught to undergraduate physiotherapy students as a normal part of their respiratory assessment. Some clinicians reported that, with more experience, it was easier to complete an assessment without detailed referral information, although often this was within an Multi Disciplinary Team/specialist environment where discussion of patients and their symptoms was possible. Increased depth and breadth of experience were keys to increasing both confidence and competence in the assessment of BPD. These specialist skills were particularly important in ruling out pathology or physiological explanation of symptoms ensuring the correct diagnostic label is assigned and other causes of symptoms fully investigated.

Table 2 provides a guide to BPD assessment based on the survey responses and FG discussions. Items recorded in the survey as used ‘always’ or ‘most of the time’ are suggested as ‘standard for all assessments’. Items used ‘sometimes’ or with a range of choices are ‘recommended for all assessment’, whereas items used ‘rarely or never’ are listed as ‘optional or with specific history’.

**Table 2:** Breathing pattern dysfunction assessment: summary

Subjective Assessment	Objective Assessment
***Standard for all Ax*** **Subjective report of symptons** Patient description of Sx (record words used by to describe Sx) Patients own awareness of breathing pattern Triggers to Sx Recovery techniques/easing factors Air hunger signs (yawning/sighing/clearing throat/tingling hands/feet)	***Standard for all Ax*** **Observation of breathing** Mouth/nose breathing Upper/lower chest Respiratory rate Air hunger Accessory muscle use Rhythm of breathing
***Recommended for all Ax*** **Sleep** Quality/duration **Social history** Family Work Hobbies **Psychological history** History of psychological illness Stress and coping mechanisms **Nasal symptoms** Blocked or runny nose Sinus pain Postnasal drip Altered sense of smell **Exercise ability** Frequency of exercise Intensity of exercise Time spent on exercise Type of exercise Sx with exercise SOB/cough/airway closure General physical activity levels	***Recommended for all Ax*** **Observation of breathing** Sounds on inspiration and expiration Inspiratory/expiratory ratio **Exercise/functional symptoms** (record method of Ax, for example, walk/ formal test/stairs) Changes to breathing pattern during Ax Work of breathing Accessory muscle use SpO_2_ HR Cough
***Optional Ax or if specific history*** **Voice/upper airway** Voice changes *for example, husky/strained/lost voice* Closure/discomfort in throat **Cough** Effectiveness/ease of clearance Triggers Dry/rattling/productive Feeling of airway closure **Sputum** Presence Colour/consistency 24 hour volume	***Optional Ax or if specific history*** **Voice** Upper airway sounds Voice quality **Cough** Nature/Type/Frequency Clearing throat **Postural assessment** ROM cervical/thoracic spine Core stability **Diaphragm assessment** Palpation Movement Core strength
**Patient reported outcome measures**	**Objective/observed outcome measures**
***Recommended assessments*** Nijmegen Questionnaire^40^	***Recommended assessments*** BPAT Score^14^
***Optional*** Short Evaluation of Breathing Questionnaire^30^ Dyspnea-12^29^ **Optional PROM for Ax of psychological factors could include:** PHQ-9, GAD-7, Hospital Anxiety and Depression scale	***Optional*** Breath Hold Ax^31^ Manual Assessment of Respiratory Motion^32^ Exercise test, for example, 6MWT/CPET/SWT/stairs

Ax, assessment; CPET, cardiopulmonary exercise test; HR, heart rate; 6MWT, six-minute walk test; PROM, patient reported outcome measure; ROM, range of movement; Sp0_2_, saturation levels; SWT, shuttle Walk test; Sx, symptoms.

## Discussion

### Nomenclature

In line with previous findings, our study suggests that the diagnosis of BPD is hampered by the lack of universally accepted terminology or methods of assessment.^11^ Historically ‘hyperventilation syndrome’ (HVS) was the most common way of describing BPD.^15^ The literature supports the idea that BPD is multidimensional, with HVS being just one type of BPD and that different individuals may display different phenotypes.^5^,^11 11^ Recent Cochrane reviews^6 12^ have called for consistency in terminology. Our questionnaire and FGs highlighted challenges in agreeing terminology. This is the first qualitative study to report clinicians’ opinions on this inconsistency. Clinicians emphasised the importance of clear nomenclature, although perhaps patients place greater value on the description and validation of their symptoms. Our study suggests an agreement from clinicians that hyperventilation is not the correct term to define all BPD and that the word ‘pattern’ is important. Consensus is now needed for an internationally recognised and adopted term.

### Language

FG discussions explored the concept of language in BPD assessment. Language used by clinicians may lead to an apathy or lack of understanding of the patient’s experience of BPD as has been shown in other chronic conditions like chronic pain.^20^ When diagnosis is unclear, this can impact on the patients experience by devaluing the condition to family, carers and other clinicians.

### BPD and breathlessness

Prevalence of BPD has been demonstrated in a number of different conditions including patients with asthma,^21^ COPD,^22^ anxiety,^23^ Postural Orthostatic Tachycardia syndrome^24^ and in post-COVID syndrome^7^ with papers commonly reporting higher breathlessness scores or reduced function/quality of life. Dyspnoea, like pain, has a multidimensional nature containing both sensory and affective components.^25^ The affective component has been regarded as a non-specific unpleasant or distressing experience of dyspnoea and is thought to be more commonly experienced in medically unexplained shortness of breath,^26^ which may represent patients with BPD. In a study of patients with medically unexplained breathlessness, the common symptoms were; the urge to breathe, affective aspects of dyspnoea, anxiety and tingling fingers.^27^ This contrasted with those with COPD and Asthma where wheezing, cough and sputum and palpitations were more common. However, there is limited understanding as to how BPD and breathlessness interlink. Further evidence is required to understand this and if there are differences in how we should assess and treat both primary and secondary BPD. The newly developed Breathe-VQ may be useful in such assessment accounting for vigilance or breathing.^28^

### The value of assessment

FG discussions described how the subjective assessment creates opportunities for patients to describe symptoms of ‘BPD’ in detail and the importance of this in validating the patients’ experience of these symptoms. This validation has an important role at the start of the therapeutic intervention, underpinning education strategies integral to breathing retraining. Moreover, the anxieties around the label of BPD may be ameliorated by such validation.

### Components of assessment

The literature describing BPD assessment suggests it lacks standardisation. Despite this, there has been an increased interest in this area with two systematic reviews registered on prospero in this subject. Many of the currently available outcome measures assess some, but not all of the different presentations of BPD.^13^ Some patient-reported outcome measures are not validated in all presentations of BPD (NQ,^15^ D-12^29^) or have limited validity (Short Evaluation of Breathing Questionniare^30^). Objective assessment tools lack sufficient evidence (BH^31^ and BPAT^14^) or are perceived to be too complex for general use (Manual Assessment of Respiratory Motion^32^). Conversely, our survey shows there are consistencies in which assessment tools are preferred by clinicians including the NQ, BH and BPAT. Our study indicates that the assessment tools are somewhat useful but not valued as central to assessment, rather they are complimentary to the expert ‘skill and art’ of the clinician. Further studies are needed to confirm validity, reliability and responsiveness to change of assessment tools in populations of individuals with primary BPD. Additionally, the majority of our responses were from B7 physiotherapists (75%) and so it may be important to evaluate individuals of different bands and different levels of experience separately to understand any differences.

Survey results indicated that respiratory rate, upper/lower chest movement, nose/mouth breathing, signs of air hunger were important components of the BPD assessment. These components, included in the BPAT, may support why there has been good clinical uptake of this tool. Additionally, discussions in the FG describe how other assessment tools including the HiLo^33^ assessment, BH and other Musculo-skeletal assessments are well used. These techniques may add additional value to an assessment, including having value for the patient to use in self-assessment during remote or virtual assessments which has had increased focus during the COVID-19 pandemic.^34^ Further research is required to determine the clinical relevance of these measures.

### The diaphragm

One area of conflict within the FG was the perceived role of the diaphragm in BPD and how this might inform both assessment and treatment. Survey results suggested that many clinicians do not routinely assess its function specifically. Dysfunction of the diaphragm is a well-described phenomenon in pathological disease, including obstructive lung disease where the diaphragm can be put at a mechanical disadvantage and neuromuscular disease when the diaphragm can be weak.^35^ However, its role in BPD is unclear. The literature suggests that the diaphragm has a dual role in both respiration and mechanical stabilisation of the spine via increased intra-abdominal pressure.^36^ This dual function must involve coordination of the diaphragm and other muscles surrounding the abdominal cavity and may compromise the respiratory motion of the rib cage and abdomen.^37^

It is unclear how diaphragmatic assessment is currently best performed, whether the diaphragm is at a mechanical disadvantage, or being used excessively to help maintain core control. Further studies are warranted.

#### Specialist skills

The FG discussion suggested a juxtaposition between the importance of normalising assessment of breathing pattern as part of all education of breathing, against the idea that a breathing pattern assessment is a specialist skill.

To our knowledge, this is one of the first qualitative clinician-focused investigations into this complex and important condition. Much of the literature has described the importance of improving the consistency of assessment and this paper offers unique insights into physiotherapists’ experiences of BPD assessment processes. Our methodology enabled detailed exploration of the components of assessments and also included the conflicts in the nomenclature used for this condition, and the different thinking around the assessment of the diaphragm. Furthermore, our survey responses from 103 participants are considered generalisable to UK physiotherapy practice, considering there are over 1500 members of the Association of Chartered Physiotherapists in Respiratory Care with only a minority of these members treating patients with BPD regularly. We also provide practical recommendations for the essential components to include in physiotherapy BPD assessment as summarised from the results in table 2.

We attempted to ensure that the questionnaire was widely accessible, including to international participants. However, there were only seven participants who identified themselves as international. This is important when generalising the results to the international community as our results may not be representative outside the UK. We also acknowledge a potential responder bias, in that those who completed the survey were more likely to have expert experience and may not be reflective of the views of more junior staff. Additionally, there were many opinions shared in the FG that alluded to a perception of how a patient may think or feel about a certain aspect of BPD assessment. However, we did not include patients within the study design, which would be an essential component for future studies.

The results emphasise the importance of a consistent approach to both terminology and assessment of this condition. Although there are some assessment tools available, there appear to be limitations to these, and further work is required to develop our knowledge with these tools to help us have a clear way of evaluating patients consistently and providing them with relevant therapeutic treatments at the earliest opportunity. Additionally, further understanding is required about how these assessments may help us to recognise the different types of BPD.^2^

Although not discussed in the FGs, recognition of different types of BPD may have importance to help ensure that the correct types of intervention are chosen.^38^ This warrants further research. Moreover, discussions have also focused on the problems in objective evaluation, but this may not be as important from the patient perspective, and, therefore, further understanding is required about patients’ experiences of having BPD and the important components of assessment.

### Conclusion

BPD is not a trivial condition. It is associated with significant morbidity and can be treated with physiotherapy. Limited evidence exists regarding how best to assess BPD. Our research combined online survey results and FG data to explore BPD assessment from clinical experts in the field. We detail the complexities of BPD assessment and remaining uncertainties. There is a clear need for consistency around the terms used, appropriate diagnostic tools and validated outcome measures. These issues could be impacting on the consistency of assessments and the adequate referral of patients to appropriate services. The themes in this study could help to direct future education, training and guidance for this condition and will help underpin the development of future research into BPD.

## Data Availability

All data relevant to the study are included in the article or uploaded as supplementary information.
