# Supplementary material for: Physiotherapy assessment of breathing pattern disorder: a qualitative evaluation
Source: BMJ Open Respir Res. 2023 Jan 10;10(1):e001395. doi: 10.1136/bmjresp-2022-001395 (PMC9835958; doi:10.1136/bmjresp-2022-001395)
Supplement: Supplementary data [file bmjresp-2022-001395supp001.pdf]

NHLI

An analysis of Breathing Pattern and its assessment by Physiotherapists working in this field

Welcome to this survey on Breathing Pattern asessment

You are being invited to take part in a research study. Before you decide it is important for you to understand why the research is being done and what it will involve. Please take time to read the following information carefully and discuss it with others if you wish.

Ask us if there is anything that is not clear or if you would like more information. Take time to decide whether or not you wish to take part.

Thank you for reading this.

What is the purpose of the survey?

Breathing Pattern Dysfunction (BPD- sometimes called 'Dysfunctional Breathing') is a condition in which people experience dyspnoea, air hunger and often limitation in function. In addition, it is often is characterised by other symptoms including tachycardia, dizziness and parathaesia. Diagnosis is difficult due to there being no gold standard terminology or method of assessment. Heterogeneity in presentation of BPD can further lead to inconsistencies in how patients are assessed and in turn treated. Evidence suggests there is modest agreement of the important components needed for an assessment; despite this, the evidence often concludes that further consensus is required. Moreover, inconsistency of terminology adds to this complexity. This survey and focus group aim to collate physiotherapists' views on the most important aspects of an assessment and gain a consensus on terminology.

With this survey we aim to evaluate physiotherapy services for BPD and to look at the components included in an assessment. This survey aims to gain an insight into the frequency of BPD assessments occurring in respiratory physiotherapy services and to look at common components included in an assessment. It will also aim to gain understanding of the preferred terminology used to describe this condition.

Why have I been invited?

You have been asked to take part as you are a physiotherapist working in BPD. We are inviting physiotherapists whom treat patients with this condition to fill in the online survey.

Do I have to take part?

Your participation is entirely voluntary. It is up to you to decide whether to take part.

Will my information be confidential?

Your responses will be collected anonymously. Please be as honest as possible as we are keen to look at responses in general and explore if further consensus building is required.

The Focus group

An *optional* second stage of this survey will occur as a focus group. This Focus Group will aim to explore the responses of this questionnaire in more detail. Please see the end of the survey for more information about the focus group.

Data Protection
Responses are collected through Survey Monkey and not identifiable to the researchers. Your data will be processed as part of Survey Monkey privacy policy which can be reviewed here (https://www.surveymonkey.com/mp/legal/privacy-policy/). As this survey is supported by imperial college, their own data protection policies will also be relevant. more information on these can be found here: http://www.imperial.ac.uk/admin-services/secretariat/information-governance/data-protection/

Thank you in advance for your input.
Version 1 4/10/19

1. Do you agree to the above terms, by clicking Yes, you consent that you are willing to answer the questions in this survey.

- ○ Yes
- ○ No

An analysis of Breathing Pattern and its assessment by Physiotherapists working in this field

Please fill in your respones as honestly as possible and feel free to expand on your responses in the 'other' boxes.

2. What is your level of experience

- [ ] Band 5 or 'newly qualified'
- [ ] Band 6 or 'rotational staff member'
- [ ] Band 7 or 'specialist physiotherapist'
- [ ] Band 8 or 'highly specialist physiotherapist'
- [ ] Other (please specify)

3. What age of patients do you see? (tick all that apply)

- [ ] Adults
- [ ] Paediatrics

4. Do you assess and treat inpatients (IP), outpatients (OP) or both?

- ○ IP
- ○ OP
- ○ Both

5. What is your place of work (tick all that apply)

- ☐ Teaching hospital
- ☐ General hospital
- ☐ Community
- ☐ Private
- ☐ Other (please specify)

6. How many patients are referred for a breathing pattern assessment to your service per week

- ○ 1 or fewer
- ○ 1-5
- ○ 5-10

7. How many patients do you assess for breathing pattern problems per week

- ○ 1 or fewer
- ○ 1-5
- ○ 5-10

8. Do you have access to a respiratory consultant/GP with a special interest in breathing pattern?

- ○ Yes
- ○ No
- ○ Other/comments

9. How confident do you feel in assessing breathing pattern (scale 1-10)

unconfident confident

10. If you have an Outpatient Service, how long do you have per assessment

- ○ 30 mins
- ○ 45 mins
- ○ 60 mins
- ○ Other

11. How often do you include the following subjective elements in your first assessment of breathing pattern

	Never	Rarely	Sometimes	Most of the time	Always
Description/discussion of onset of the breathing pattern symptoms	○	○	○	○	○
Discussion of triggers for breathing pattern symptoms	○	○	○	○	○
Description/discussion of what makes their breathing pattern symptoms worse/better	○	○	○	○	○
Nose/sinus Symptoms	○	○	○	○	○
Cough/throat symptoms	○	○	○	○	○
Voice symptoms	○	○	○	○	○
Discussion about how your patient manages their breathing pattern symptoms?	○	○	○	○	○
Symptoms of breathing pattern with activity/exercise	○	○	○	○	○

Please describe any other elements that you routinely include in your assessment

12. How often do you include the following objective elements in your first assessment of breathing pattern

	Never	Rarely	Sometimes	Most of the time	Always
Respiratory Rate	○	○	○	○	○
Method of breathing: nose/mouth	○	○	○	○	○
Upper Chest v Lower chest movement	○	○	○	○	○
Erratic/rhythmical breathing	○	○	○	○	○
Air hunger frequency (sighs/yawns)	○	○	○	○	○
Sounds or flow on inspiration and expiration	○	○	○	○	○
Inspiration/expiration ratio	○	○	○	○	○
Breath holding	○	○	○	○	○
Accessory muscle use	○	○	○	○	○
Cough/throat clearing	○	○	○	○	○
Breathing Pattern observation on movement (walking, stairs, steps, exercise)	○	○	○	○	○
Diaphragm palpation	○	○	○	○	○
Musculoskeletal Ax (this may include posture, thoracic spine assessment etc.)	○	○	○	○	○

Please describe any other elements that you routinely complete as part of your assessment

13. Please tick all the subjective assessment tools you complete in your assessment

- [ ] Short Evaluation of Breathing Questionnaire (SEBQ)
- [ ] Dyspnea-12
- [ ] BORG scale
- [ ] Nijmegen Questionnaire
- [ ] Hospital Anxiety and Depression Scale (HAD)
- [ ] None
- [ ] Other (please specify)

14. Please tick all the objective assessment tools you complete in your assessment

- [ ] Breath hold
- [ ] End Tidal CO2
- [ ] Exercise test e.g. 6MWT/SWT/Step Test
- [ ] Manual Assessment of Respiratory Motion (MARM)
- [ ] Brompton Breathing Pattern Assessment Tool (BPAT)
- [ ] None
- [ ] Other (please specify)

15. Do you have access to any of the following assessments at your place of work

- [ ] Cardiopulmonary exercise testing (CPET)
- [ ] Continuous laryngoscopy during exercise (CLE)
- [ ] Provocation Laryngoscopy
- [ ] Lung Function (spirometry)
- [ ] Other (please specify)

16. On average, how many sessions do your patients complete

- ○ 1-2
- ○ 2-4
- ○ 4+

17. Are you referred patients with Inducible Laryngeal Obstruction (ILO) or Exercise Induced Laryngeal Obstruction (EILO)?

- Yes
- No
- Don't know

18. How confident do you feel in **assessing ILO** (scale 1-10)

Unconfident — Confident

19. How confident do you feel in **treating ILO** (scale 1-10)

Unconfident — Confident

20. How confident do you feel **assessing EILO**

Unconfident — Confident

21. How confident do you feel **treating EILO**

Unconfident — Confident

22. How do you most frequently manage patients with ILO (tick all that apply)

- [ ] I refer to a Speech and Language Therapist with an interest in Upper Airway (UA SLT) in my trust/department
- [ ] I refer to an UA SLT outside of my trust/department
- [ ] I see ILO patients jointly with an UA SLT
- [ ] We treat ILO in our physiotherapy service
- [ ] Other (please specify)

23. How do you most frequently manage patients with EILO

- [ ] I refer to an UA SLT in my trust/department
- [ ] I refer to an UA SLT outside of my trust/department
- [ ] I see ILO patients jointly with an UA SLT
- [ ] We treat EILO in our physiotherapy service
- [ ] Other (please specify)

24. Do you have access to a psychologist?

- ○ Yes
- ○ No
- ○ Other (please specify)

25. Would you be willing to take part in a focus group to explore these responses in more detail. This will be held in central London on the 14th November 1000-1400. An honorarium towards travel will be paid.

If you indicate yes, please email l.grillo@imperial.ac.uk to confirm your interest.

- ○ Yes
- ○ No

26. Thank you for completing this questionnaire.
One final question; What is your preferred term for this condition?

- ○ Hyperventilation
- ○ Dysfunctional Breathing
- ○ Breathing Pattern Disorder
- ○ Breathing Pattern Dysfunction
- ○ Other (please specify)
