# Supplementary material for: Physiotherapy assessment of breathing pattern disorder: a qualitative evaluation
Source: BMJ Open Respir Res. 2023 Jan 10;10(1):e001395. doi: 10.1136/bmjresp-2022-001395 (PMC9835958; doi:10.1136/bmjresp-2022-001395)
Supplement: Supplementary data [file bmjresp-2022-001395supp002.pdf]

Supplementary information

Methodology info

Email/Social Media recruitment for Survey and Focus group

Email to 'ACPRC' and 'Physiotherapy for Breathing Pattern'

To………

Title of Study: An analysis of Breathing Pattern and its assessment by physiotherapists working in this field

My name is Lizzie Grillo and I am an advanced Physiotherapist working clinically at the Royal Brompton Hospital within the Adult Specialist Respiratory Medicine Team. Additionally, I am undertaking a Pre-Clinical Academic Fellowship (PCAF) as part of the NIHR ICA Programme. As part of my PCAF award I am completing a project looking at Dysfunctional Breathing Assessment. The title of this study is: "**An analysis of Breathing Pattern and its assessment by physiotherapists working in this field".** This project is in two parts, the first a survey to be completed by physiotherapists whom undertake BPD Assessments, and the second a focus group to be completed after analysis of the survey to understand some of the responses and opinions within this area in more detail. I would be grateful if you could share this survey via your newsletters and social media. The survey has details of the focus group within it where you can indicate if you are able to attend. It would be great if one of your committee could also attend the focus group to ensure your organisation is represented. Please could you let me know if this is possible?

This project has been supported by Imperial College ethics committee. Please let me know if you need any more detail.

Lizzie Grillo

Information for the Newsletter/Social Media

My name is Lizzie Grillo and I am an advanced Physiotherapist working clinically at the Royal Brompton Hospital within the Adult Specialist Respiratory Medicine Team. Additionally, I am undertaking a Pre-Clinical Academic Fellowship (PCAF) as part of the NIHR ICA Programme. As part of my PCAF award I am completing a project looking at Breathing Pattern Dysfunction (BPD) Assessment. The title of this study is: "**An analysis of Breathing Pattern and its assessment by physiotherapists working in this field".** This project is in two parts, the first a survey to be completed by physiotherapists whom undertake BPD Assessments, and the second a focus group to be completed after analysis of the survey to understand some of the responses and opinions within this area in more detail. If you are a physiotherapist (of any level of experience) whom completes BPD Assessments, I would be grateful if you could spend 5-10 minutes this survey (insert link). The survey has details of the focus group within it where you can indicate if you are able to attend.

This project has been supported by Imperial College ethics committee. Please let me know if you need any more detail.

Lizzie Grillo

Follow up emails

To………

This is a follow up email to my original email dated (insert date of first email). My name is Lizzie Grillo and I am an advanced Physiotherapist working clinically at the Royal Brompton Hospital within the Adult Specialist Respiratory Medicine Team. Additionally, I am undertaking a Pre-Clinical Academic Fellowship (PCAF) as part of the NIHR ICA Programme. As part of my PCAF award I am completing a project looking at Dysfunctional Breathing Assessment. The title of this study is: "**An analysis of Breathing Pattern and its assessment by physiotherapists working in this field".** This project is in two parts, the first a survey to be completed by physiotherapists whom undertake BPD Assessments, and the second a focus group to be completed after analysis of the survey to understand some of the responses and opinions within this area in more detail. I would be grateful if you could share this survey via your newsletters and social media. The survey has details of the focus group within it where you can indicate if you are able to attend.

This project has been supported by Imperial College ethics committee. Please let me know if you need any more detail.

Lizzie Grillo

Focus Group email (individuals)

To…..

Thank you for your interest in attending the Focus Group for the study: "**An analysis of Breathing Pattern and its assessment by physiotherapists working in this field".**

I have attached the participant information sheet for you to read. Please take time to read this and I invite you to ask any questions prior to agreeing to attend.

The focus group will be held at Imperial College, Emmanuel Kaye Building, Manresa Road on (insert date) from 1000 until 1400. You will be provided with lunch, so please indicate any dietary requirements. We will also be providing an honorarium for up to £60 for your travel on the day.

Please confirm via email if you can attend and you will receive further information about the day.

Thank you in advance for your time.

Focus Group (specific group email)

To ACPRC/Physiotherapy for BPD (delete as appropriate)

Thank you for providing a representative to attend the Focus Group for the study: "**An analysis of Breathing Pattern and its assessment by physiotherapists working in this field".**

I have attached the participant information sheet for you to read. Please take time to read this and I invite you to ask any questions prior to agreeing to attend.

The focus group will be held at Imperial College, Emmanuel Kaye Building, Manresa Road on (insert date) from 1000 until 1400. You will be provided with lunch, so please indicate any dietary requirements. We will also be providing an honorarium for up to £60 for your travel on the day.

Please confirm via email if you can attend and you will receive further information about the day.

Thank you in advance for your time.

Demographic information

We collected 103 responses. 75% or responders were a Band 7 or higher (highly specialist physiotherapists), 20% B6 (specialist physiotherapists) or below and 5% other (lecturer practitioner, private physiotherapists). 65% saw both inpatients and outpatients, with 25% just seeing outpatients alone. 57% worked in a tertiary or teaching hospital, 25% in secondary care, 25% in primary care and 14% privately. There was a range of experience based on patients seen per week with approximately 1/3 seeing less than one patient with BPD per week, 1/3 seeing 1-5 patients with BPD per week and 1/3 seeing more than 5 patients with BPD per week.

Focus Group Topic Guide

Question	Prompts
What is your preferred terminology for describing Breathing Pattern problems?	Likes/Dislikes of terms<br>Problems with variation of nomenclature
What is BPD	BPD v Breathlessness<br>Diagnosis v assessment
What are the important components of a Breathing Pattern Assessment?	Subjective Assessment components<br>Objective assessment components<br>Assessment Tools<br>How do you diagnose BPD
Can you discuss the role of exercise (assessment) in BPD	Limitation to including exercise assessment<br>What components of assessment do you include
Describe an optimal assessment of BPD	Skills required<br>Equipment required<br>Barriers/enablers

Table 5: Additional Quotes from the Focus groups

**Nomenclature and Language**
I think it is frustrating, the terminology. It doesn't help does it, in us trying to help diagnose, doing research, you know having outcome measures, when we sort of don't even have this start point (R2)
"it is highly important to define and be clear going forward with what the condition is and how it is described." (R7)
"But it is what we want to define it for. Is it useful for us to define it, or is it useful for the patient? And I guess maybe that is where we are having a bit of a problem. I don't know, but that is how it feels, that we are trying to get it the best, but for whom?". (R4)
'I don't really mind what it is called because I think the art of being a good clinician is then to follow it up with an explanation in lay terms that buys the patient in to what you are going to teach them'. (R1)
"Patients can often come in thinking "I have done all this stuff, and everybody says there is nothing wrong with me." And I say, "Actually, you are just not breathing well. You're okay, but you're not crazy." And I think a lot of patients think, "They all think it's in my head." (R4)
'There are a lot of patients like this that have kind of got themselves a bit of a reputation, and as clinicians we have kind of earmarked them as that, "Ah, they are probably breathing pattern' (R9)
They both (BPD and DB) surmise a myriad of different altered presentations of breathing leading to a range of symptoms. But hyperventilation is a symptom, not the cause as such. (R4)
But I agree, hyperventilation isn't a useful term, I don't think. It doesn't actually describe a lot of our patients. (R2)
"I think patients often arrive with terms themselves don't they, they have been told things like hyperventilation. I think often patients don't label themselves even after treatment with me, do they? They don't go around saying, "I have a breathing pattern disorder," like people often do with COPD or asthma, they will like label themselves" (R12)
So, I think we are nit-picking, and language is a problem, but it is working out who we are aiming the language at really and how the doctors describe it to patients in order to get them to engage with physio, and it is a minefield (R7)
"I have had a few patients that have wanted the diagnosis. When the original asthma diagnosis has been questioned and their asthma has been down on a piece of paper to say they can't work, then you take that away and what have they got? Because their breathing pattern could be so horrendous that that is a barrier for them doing a normal job, and you are working with them to overcome that" (R15)
"I think it is also important to align it with national and international consensus" (R16)
"I always say, "You can use any language you like." Because a lot of my patients won't have technical language, so I say to them, you know people will say to me, "I feel like I have got a double decker bus parked on my chest." So, I think, "Maybe that is chest tightness." It's about allowing them to use whatever language they like" (R17)
"There is a lot of patients like this that have kind of got themselves a bit of a reputation, and as clinicians we have kind of earmarked them as that, "Ah, they are probably breathing pattern." (R10)
**BPD & Breathlessness**
"So, it's maybe a large group of symptoms that do not necessarily fit in with any other standardised or recognised patterns of breathlessness, and are not responsive to certain conditions, medications, or management" (R5)
"If you physiologically think of breathlessness, it is a combination of insufficiency of gas exchange at some level, whether it is at the lung or the cell, it doesn't matter. So they come to you and say, "I don't feel that I can get enough breath. What do I call that? I am breathless." (R7)
Personally I spend quite a lot of time asking patients to describe symptoms and trying to work out what is it that actually is bothering them? And sometimes it is just 'I have become really aware of my breathing'. So, there is a whole kind of range from mechanical type symptoms to a kind of cognitive awareness, but it gets sort of put under this umbrella or breathlessness when they first some to see you" (R3)
'Breathing is not just a sensation it is an emotion' (R1)
**Diagnosis and validation of symptoms**

“When we have done our assessment this not only helps us diagnose it but it also shows us that they are breathing too fast or their breathing’s irregular. The causes are something different and the treatment is something different, but you are diagnosing BPD.” (R13)
“And it was weekly that someone would come and sit down and cry, that’s the first thing they did, because they felt that they were wasting everyone’s time, and that they were being blamed for whatever was going on, and that they were sitting in a clinic with people who were genuinely sick, but their symptoms were so difficult” (R8)
“I think very much like you said, the issue is, is this breathlessness in excess or breathlessness in the absence of a diagnosis? And 99% of what I see is the two together, there is at least one diagnosis underlying” (R14)
“But I think there is such a roll of it happening in primary care before … and that is I don’t think where we are getting involved enough yet, and I think that would be a really exciting place that this could be picked up earlier, alongside other potential investigations” (R10)
**Components of Assessment**
“I think the subjective is probably most important, you are trying to understand the long and winding road that they have taken to get to you.” (R1)
“I think probably the most transformative thing that we have had in our service, that I am sure many of you have, is just electronic record sharing. It is amazing, because the relevant stuff is often what you are searching for very specifically. And we are on the same record as the GPs, so you get all sorts of mental health history that no-one would have asked the patient about, that becomes super relevant for your assessment” (R5)
**Assessment Tools**
“I’m not saying they are not useful. I’m saying we need to be mindful of the fact that we are on a journey with all these outcome measures and it is certainly not the finished article” (R11)
“The NQ is obviously centred around HVS, but I find I am not really bothered about the result, but the patients find it really powerful. It often pools together some symptoms and brings out symptoms they wouldn’t have necessarily thought were related, and to be able to say, “Oh, yes that’s me, that’s me,” and they get quite excited” (R8)
I think if you have been doing it for a very long time and you have seen many patients, a lot of that is happening anyway, you are just putting in what you were already doing and thinking about into a structural form”. (R11)
“I could quite happily not use any of the outcome measures that are available at the minute and just look at the patient and how they are breathing and assess their breathing pattern and then put your hands on the patient. I think that gives us far more information than any of the written stuff at the minute” (R17)
“ I think there is a real dangerous route that we could potentially go down where we are trying to show value in the service that we are providing based on measures that aren’t fully” (R11)
“Although there is limited data in the (clinical) value of the breath hold, it is a quick, easy assessment to complete- often improving with treatment and therefore providing important feedback to both the patient and clinician through recovery”. (R12)
**Diaphragm**
“The diaphragm is hardly contracting at all-, so it is fixed and holding onto all that air”. (R8)
“So, I will often get patients and the way that I can kind of, I call it the hook, is I will lie them on the bed, and I will say to them, “I want you to let go,” and their diaphragm will kick in and they will go, “That feels so much better.” And I’m like, “Right, that’s what you need to get” (R13)
**BPD and Exercise**
“Quite often when they come to do the exercise bit it either all goes pear-shaped and you have to address it very specifically, or actually they morph into it quite naturally because you have improved their breathing pattern, therefore they go on their demand, not what they think their breathing pattern should be.” (R4)
**Specialist Skills**
‘I think you need a certain level of testing. I think the best referrals I get are from the respiratory consultants. This can make the assessment easier at the beginning and help with decision making’ (R1)
“I like to know what their label is, and what tests have been ran, and what has been excluded. The more experience you have, the more speedily you can filter the information”. (R13)
‘If you have been referred someone with the diagnosis of BPD, it shouldn’t stop you being curious about something else going on’ (R14)

'I think we have got to be really careful of presuming BPD, or presuming dysfunctional breathing, and I am very cautious that we don't have a culture where we are so quick to promote that BPD exists that we want to label people with it. I think we have to be really careful…. making sure that those comorbidities are properly managed within a label of BPD'. (R8)
"perhaps these people don't have the exposure or the skills to be able to pick up on small nuances that might be wrong to be able to tweak things in the management" (R15)
"And that is an art I think, and that is where the experience of specialism comes in, where you have just seen, and tried, and done, and thought about it in a different way, and put it in a slightly different way, and it has worked for that one or two people, and you have got another skillset" (R17)
